# Nothing in modern biology makes sense except in the light of ecology and biodiversity conservation

**DOI:** 10.1093/conphys/coy025

**Published:** 2018-05-23

**Authors:** David Costantini

**Affiliations:** 1 UMR 7221 CNRS/MNHN, Muséum National d’Histoire Naturelle, Sorbonne Universités, 7 rue Cuvier, Paris 75005, France; 2 Department of Biology, University of Antwerp, Universiteitsplein 1, Antwerp 2610, Belgium; 3 Institute of Biodiversity, Animal Health and Comparative Medicine, University of Glasgow, Glasgow G12 8QQ, UK

## From the field to the lab… and back

‘Nothing in Biology Makes Sense Except in the Light of Evolution’ is a 1973 essay by the evolutionary biologist Dobzhansky (Dobzhansky, 1973). This classical essay has been dramatically influential in fighting the anti-evolution movement and in developing the discipline of evolutionary biology further. But nowadays we live in a moment in history where disciplines like ecology and biodiversity conservation are playing a central role in the life sciences. Opportunities for studying truly natural processes are diminishing because pristine or near-pristine environments are rapidly shrinking and becoming more difficult to reach. This reminds us that we need to conceptualize and contextualize research in the framework of global environmental change to learn how organisms respond to life in the ‘Anthropocene’. This knowledge is fundamental to develop successful conservation programmes.

I grew up as a naturalist and field biologist with a strong interest in physiology. Particularly, I have been a pioneer in the fledgling fields of oxidative stress and antioxidant ecology. Research on and funding of these topics have, however, been historically centralized on a few model laboratory organisms. This has driven me to explore the physiological work on animals housed in captivity. I have appreciated the strong potential of captivity-oriented work for conducting experiments under controlled conditions to answer very specific mechanistic questions. I have also seen, however, strong limits in laboratory-oriented physiological work, especially when stepping into the wild to assess whether laboratory-based results apply to the real world. The main pillars of physiology have been human, mouse and a handful of other organisms (e.g. *Drosophila*, *Caenorhabditis elegans*, rainbow trout). In these settings, investigators seek to reduce complexity and control environmental conditions. However, the world outside the laboratory is immensely variable, offering a plethora of opportunities to explore the secrets of life diversity and to apply such knowledge to conservation of biodiversity and sustainable wildlife management. Thus, in modern biology nothing makes sense except in the light of ecology and biodiversity conservation. Clearly, my statement is provocative: it reminds us the central roles that these disciplines have in modern life sciences.

At a certain point in my career, I thought the time was ripe to go back to the field-based work, bringing physiological knowledge and tools into the real world. I also thought that tropics would have been an ideal starting point. Tropical organisms and ecosystems are facing great challenges that make conservation a priority. Wood harvesting and conversion of logged forests to agricultural lands are key drivers of the global extinction crisis in the tropical areas. Expansion of agricultural lands is also causing a global concern because of the use of pesticides (even nasty chemicals banned in Europe or North America) to reduce the prevalence of pest species. Environmental contamination in the tropics also comes from other sources. For example, tropical freshwater and marine ecosystems are under serious threat due to widespread contamination with mercury, used for the extraction of gold in mines made in forests. Thus, over the last few years, I have travelled widely in the tropics, aiming to use physiological tools to assess and, possibly, predict the extent to which organisms are responding to the ongoing environmental changes.

## Seabirds in Amazonian ecosystems

Since 2005, a high nestling mortality, associated with visible nodular proliferative skin lesions, has been regularly recorded in a colony of Magnificent frigatebirds (*Fregata magnificens*) breeding on the Ile du Grand Connétable (French Guiana, 4° 49′ N; 51° 56′ W). Histopathological investigations of carcasses showed that nestlings were in poor nutritional condition, were free of bacterial infections or ectoparasites, were negative for avian poxvirus DNA, but were positive for herpesvirus DNA (de Thoisy *et al.*, 2009).

On the Ile du Grand Connétable, the diet of adult frigatebirds seems to be very dependent on discarded fishes from the shrimp trawlers. Interestingly, the activity of industrial shrimp trawling fisheries had decreased for economic reasons around 1 month before the first symptoms of the viral disease were recorded in nestlings (de Thoisy *et al.*, 2009; Martinet and Blanchard, 2009). In recent years, there has also been a dramatic increase of mercury contamination of freshwater and marine South-American ecosystems (Laperche *et al.*, 2014). This has raised a growing concern about the effects of mercury on both wildlife and human health, particularly in French Guiana (where viral outbreaks in frigatebirds are occurring), which is one of the regions that is experiencing the most dramatic increases of mercury contamination. I thought this would have offered an exciting opportunity to start a new research project aimed to identifying the environmental causes and patho-physiological consequences of viral outbreaks in a seabird population of conservation value. This kind of project clearly needed a multidisciplinary team. I have therefore involved several colleagues and institutions with complimentary expertise, including ecotoxicology (Olivier Chastel, CNRS; Paco Bustamante, La Rochelle University), behavioural endocrinology (Marcel Eens, University of Antwerp), telomeres (Frédéric Angelier, CNRS), virology (Vincent Lacoste and Benoît de Thoisy, Institut Pasteur de la Guyane), monitoring and management of the island (Kévin Pineau, Groupe d’Etude et de Protection des Oiseaux en Guyane). And, importantly, the project also needed of a talented and motivated Ph.D. student (Manrico Sebastiano, University of Antwerp) capable in fieldwork, but also in helping with the coordination of such an ambitious project.

Manrico Sebastiano, colleagues and I have performed several experiments to determine the physiological and immunological differences between healthy and sick nestlings and to identify possible environmental sources of stress that favour the viral replication. Although the work has not been finished yet, data collected so far have already allowed us to either identify or discard potential factors relevant for the progress of the disease. For example, nestlings with severe clinical signs of the disease suffer higher molecular oxidative damage and inflammation than those not showing any clinical signs (Sebastiano *et al.*, 2017b,c). Markers of oxidative damage and inflammation were also significantly associated with the short-term probability of survival of nestlings. These findings have been important because physiological markers change faster than clinical signs do. Thus, given their association with the disease, those markers might be used to assess the efficiency of a pharmacological treatment.

One of the main questions of the project that remains unanswered is which environmental factors cause the viral outbreak. Observations done by us and colleagues in other frigatebird populations in central and South America suggested that these viral outbreaks might be very specific of the population breeding on the Ile du Grand Connétable. This indicates that these frigatebirds are being exposed to a (or multiple) local source of stress that could originate from the collapse of local fisheries (nutritional stress hypothesis) or from the environmental contamination (contamination stress hypothesis). Our work showed that mercury occurs in blood of frigatebirds at concentrations that (i) exceed those considered to be of low risk for the individual health and (ii) are much higher than in other seabird species breeding sympatrically (Sebastiano *et al.*, 2016, 2017a). Conversely to mercury, other metallic pollutants or persistent organic pollutants occurred at undetectable to low concentrations (Sebastiano *et al.*, 2016, 2017a). This result has raised the important question of whether mercury favours pathological statuses in frigatebirds. Interestingly, clinical signs similar to those expressed by frigatebird nestlings were observed in a few nestlings of laughing gull (*Leucophaeus atricilla*) and Royal tern (*Thalasseus maximus*), which have the higher blood mercury concentration on the island after that of frigatebirds. Based on the already available results that pointed out extreme physiological differences between healthy and sick birds, we have very recently carried out experiments aimed to manipulating specific aspects of the physiological status of nestlings, such as the intake of nutrients or of antioxidants. These experiments will help to identify which factors favour viral outbreaks. This information will also be relevant for planning an effective conservation programme of this frigatebird population, which is one of the most important in South America.

## Conclusions

Using a combination of multiple approaches (such as those described above) in which physiology plays a central role, I hope that we can better elucidate the causes and consequences of viral outbreaks in frigatebirds, as well as in sympatric seabirds, and use this information to inform policy makers and natural resource managers to make the best decisions to preserve these seabird populations and the environments they live in. As field biologists wearing hats such as ecologist, environmental physiologist and conservation physiologist, we should not lose sight of one extremely important concept proposed by Noss (1996): ‘Nothing will destroy the science and mission of conservation biology faster than a generation or two of biologists raised on dead facts and technology and lacking direct, personal experience with Nature.’ The Anthropocene demands that the scientific community and allied partners and stakeholders tackle the many challenges facing the natural world. Fieldwork is not better than lab work or vice versa—they are complementary. I urge biologists to consider working in both realms and in the space in between (e.g. field mesocosms) to ensure that the work we do is relevant (see Cooke *et al.*, 2017) to solving the problems facing biodiversity in the Anthropocene.

**Figure:**
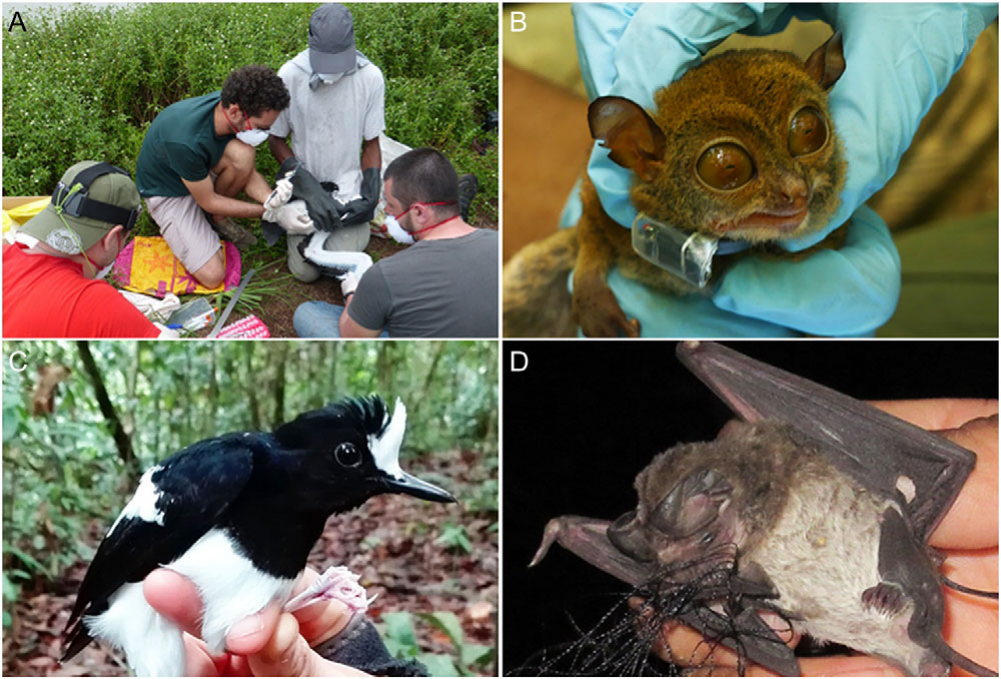
David Costantini received his PhD from the University of Rome ‘La Sapienza’ and is currently a full professor at the Muséum National d’Histoire Naturelle in Paris. Over the last few years, he has travelled widely in the tropics, where countries are rapidly transforming pristine habitats into anthropogenically shaped and contaminated landscapes. His goal has been to bring laboratory-based physiological knowledge and tools into the real world to address questions of ecological and conservation relevance: (**A**) links among environmental contamination, food limitation and viral diseases in Magnificent frigatebirds (*Fregata magnificens*) and other seabirds in Amazonian coastal ecosystems; (**B**) behaviour, energetics and trophic ecology of nocturnal primates in fragmented forests in Malaysian Borneo; (**C**) physiological responses of forest birds to logging in Malaysian Borneo; and (**D**) stress physiology of wrinkle-lipped free-tailed bats (*Chaerephon plicatus*) across landscapes with different degrees of agricultural impact in Thailand.
